# Cultural Variation in the Effectiveness of Feedback on Students’ Mistakes

**DOI:** 10.3389/fpsyg.2019.03053

**Published:** 2020-01-21

**Authors:** Kimmo Eriksson, Jannika Lindvall, Ola Helenius, Andreas Ryve

**Affiliations:** ^1^School of Education, Culture and Communication, Mälardalen University, Västerås, Sweden; ^2^Centre for Cultural Evolution, Stockholm University, Stockholm, Sweden; ^3^NCM, University of Gothenburg, Göteborg, Sweden

**Keywords:** negative feedback, power distance, religiosity, cultural values, effective instruction, mistakes

## Abstract

One of the many things teachers do is to give feedback on their students’ work. Feedback pointing out mistakes may be a key to learning, but it may also backfire. We hypothesized that feedback based on students’ mistakes may have more positive effects in cultures where teachers have greater authority over students, which we assume to be cultures that are high on power distance and religiosity. To test this hypothesis we analyzed data from 49 countries taking part in the 2015 wave of the TIMSS assessment, in which students in the 4th and 8th grades were asked whether their teachers in mathematics and science told them how to do better when they had made a mistake. For each country we could then estimate the association between the reported use of mistake-based feedback and student achievement. Consistent with our hypothesis, the estimated effect of mistake-based feedback was positive only in certain countries, and these countries tended to be high on power distance and religiosity. These results highlight the importance of cultural values in educational practice.

## Introduction

Some scholars argue that pedagogical methods and concepts are culturally embedded and that transplanting them from one culture to another is not always feasible (e.g., Hatano and Inagaki, 1998; Chen and Wong, 2015). In the present paper we focus on cultural differences in the effects of a specific teacher practice: to give feedback on students’ mistakes. Students’ mistakes have been argued to play a key role for learning (Mangels et al., 2006; Boaler, 2016) and reform initiatives in mathematics education (National Council of Teachers of Mathematics, 2000), research within the field of mathematics education (e.g., Kazemi and Stipek, 2001; Bray, 2011) as well as studies in psychology and neuroscience (see Boaler, 2016) emphasize that capitalizing on students’ mistakes may be a particularly productive teaching practice. On the other hand, to go from mistakes to learning is not straightforward. Feedback on mistakes, also known as corrective feedback, may even be counter-productive, for example if students perceive they cannot understand the feedback, if it makes them focus on right and wrong answers instead of the solving process, or if it makes them dwell on their mistakes (Gagné et al., 1987; Fyfe and Rittle-Johnson, 2017). The timing and character of corrective feedback may therefore influence its efficacy. In general, feedback are thought to work through cognitive, motivational and meta-cognitive mechanisms that are affected by the relationship between the learning situation and the learner, and by the level of expertise and experience of the learner and the teacher (Hattie and Timperley, 2007; Harks et al., 2014). This means that social and cultural factors that influence the learning context may affect the efficacy of feedback. The purpose of the present paper is to examine the cultural dependence of the efficacy of mistake-based feedback.

### Cultural Variations in Mistake-Based Feedback

In classroom practice, teachers must choose how to react to students’ mistakes. The reaction could be anything from simply ignoring mistakes to making them starting points for whole-class discussions (Bray, 2011). Teachers’ feedback on how to do better when students have made a mistake is a particularly interesting teaching practice to study. Such corrective feedback can take many forms. For example, the teacher can criticize the student for making a mistake or praise the thought and emphasize the learning potential (Tulis, 2013), or the teacher can give feedback in the form of statements (e.g., giving the correct answer or providing an explanation) as well as questions (e.g., redirecting the question or asking for student explanation; Schleppenbach et al., 2007). Also how teachers’ feedback on errors is perceived by students may differ. For example, students can feel embarrassed when their teacher point out errors or view them as opportunities to improve (Tulis, 2013).

The ways in which teachers handle mistakes and how mistakes are perceived by the students may be nationally embedded and have shown to differ between countries. For example, in analyzing teacher–student interactions surrounding students’ mistakes in 60 videotaped 8th grade mathematics lessons, Santagata (2004) found differences between Italian and United States teachers. While United States teachers tended to mitigate student mistakes and students rarely blamed themselves for making them, Italian teachers more often aggravated student mistakes (e.g., by openly showing their disappointment) and students took responsibility for making them. Another example is Schleppenbach et al. (2007) who analyzed videotaped lessons from elementary mathematics lessons and found differences in how mistakes were treated in the United States and China. Their results indicate that United States and Chinese students made errors at similar frequencies, but that the teachers in the two countries responded to them differently. The United States teachers made more statements about errors than the Chinese teachers, who instead asked more follow-up questions about errors. Moreover, when questions were used, the United States teachers tended to question students by asking them to evaluate the answer, while the Chinese teachers questioned students by asking them to correct or explain the error. Cultural differences in how students mistakes are viewed have also been discussed by Stevenson and Stigler (1992) and Wang and Murphy (2004) who argue that, in the American context, mistakes are often seen as failures and something that makes students appear silly, while they in China and Japan are viewed as signs of what needs to be learned. Video analysis combined with student questionnaires confirm the existence of culturally dependent feedback effects in comparisons of Switzerland and Germany, where the Swiss students rate their opportunities to learn from errors higher (Dalehefte et al., 2012). Although all of these studies lay important groundwork for using a cross-cultural approach when looking at feedback on students’ mistakes, we know of no previous cross-cultural work in education focusing on the *effects* of teachers’ mistake-based feedback on student achievement at a larger-scale.

### How Culture May Influence the Effectiveness of Mistake-Based Feedback

In an influential review of how culture may influence students’ approaches to learning, Littlewood (1999) focused on aspects in which East Asian culture tends to differ from Western culture: high power distance (normalcy of inequality in power and authority) and high collectivism (emphasis on interdependence instead of individuality), including a belief in the adaptability of individuals through effort. These aspects may both have bearing on the effect of negative feedback. Psychological research has suggested that Japanese are more willing than North Americans to accept negative feedback and try to improve from it (Heine et al., 2001a, b). The theoretical rationale for this difference, according to Heine et al. (2001a, p. 435), is that Japan is a culture that emphasizes “hierarchy and role mastery” and that in this context, “the discovery of negative features about the self serves to highlight where one needs to make efforts so as to move toward the consensually shared standards.” In other words, the authors simultaneously appealed to power distance (hierarchy) and collectivism (consensually shared standards to which individuals can adapt through effort). On the basis of this prior research we may hypothesize that mistake-based feedback from teachers leads to better student achievement specifically in cultures that are high on power distance and high on collectivism. We shall now elaborate on the rationale for these hypotheses.

#### Teacher Authority, Power Distance, and Religiosity

According to Hofstede (2001), societies vary in the extent to which inequality in power is accepted and regarded as normal. When applied to the teacher-student relationship, high power distance implies that teachers have great authority. Students respect the teacher, they appreciate that the teacher tells them what to do, they speak up only when invited, and they do not contradict the teacher. Students in societies with low power distance have less respect for teachers and are more likely to challenge teachers’ authority and rely on their own experience instead (Hofstede, 1986; Woodrow and Sham, 2001; Joy and Kolb, 2009; Holtbrügge and Mohr, 2010). It would fit with this general picture of teacher authority that students would be more accepting of negative feedback from the teacher^1^.

Although we will use power distance as a proxy for cultural differences in teacher authority, we acknowledge power and authority are not exactly synonymous. An important aspect of authority is being a “reliable guide as to how things are” (Raz, 1990, p. 2), thus connecting authority with religion. It stands to reason that more religious societies are more accepting of religious authority and, plausibly, of teacher authority in general. This notion does not seem to be well researched, but scholars have argued for a strong parallel between religious authority and teachers’ authority (Smith, 2013). Another supporting piece of evidence is that country-level religiosity and power distance are strongly, but not perfectly, correlated (Parboteeah et al., 2008). For these reasons we shall use country-level religiosity as an alternative proxy for teacher authority to complement power distance.

#### Collectivism and the Interdependent and Adaptable Self

The cultural dimension of collectivism vs. individualism concerns the degree to which individuals are first and foremost regarded as parts of a collective and perceive themselves as interdependent (Markus and Kitayama, 1991; Hofstede, 2001). Compared to people in individualist countries, people in collectivist countries seem to be more likely to view ability and wisdom as adaptable through effort rather than fixed in an individual (Heine et al., 2001b). This may further be connected to the concept of fixed (believing intelligence is as fixed entity) versus growth (believing intelligence is malleable) mindset (Dweck, 2006), which have also been indicated to be associated with trait use and could help account for cultural differences (Dweck et al., 1995). In a recent meta-analytic review, for example, Costa and Faria (2018) found that educational studies conducted in Asia and Oceania reported a significant association between growth mindset and student achievement while, in Europe, a fixed mindset was modestly and positively associated with student achievement. The authors suggest that this may reflect cultural differences were more collectivist societies (such as many Asian countries) might encourage students to value the learning process over individual academic achievement, while in Europe there is a tendency toward a more academically competitive society where students may prioritize individual results over knowledge.

Moreover, it has been suggested that collectivism may facilitate the acceptance of negative feedback because it enables individuals to identify their weaknesses in order to improve and blend in Heine et al. (2001b) and Gelfand et al. (2002). As Heine et al. (2001a, b), a sense that ability is not innate but improves with effort may make negative feedback less threatening and thus presumably more effective in promoting learning. In fact, studies in neuroscience (Mangels et al., 2006; Moser et al., 2011) have shown that students react differently to negative feedback depending on differences in their mindsets. Compared to students endorsing a more fixed mindset, more growth minded individuals showed superior knowledge gains in that they demonstrated greater remediation of errors and were more likely to reflect awareness of and allocation of attention to mistakes.

### The Present Study

The aim of the present study is to examine the relation between the above-mentioned cultural dimensions and the effectiveness of teachers giving mistake-based feedback to students. Every country then provides just a single data point. It is therefore imperative to obtain data from as many countries as possible. We use data on student achievement and teaching practices in 49 countries obtained from TIMSS, the Trends in International Mathematics and Science Study. TIMSS is conducted every four years by IEA (International Association for the Evaluation of Educational Achievement). Entire classes in grades 4 and 8 are sampled and participating students are linked to the teacher/classroom level. Students are given achievement tests in mathematics and science as well as a background questionnaire including some items on teachers’ use of various instructional practices. Such data can be related to student outcomes to estimate the association between instructional practices and achievement (e.g., Blömeke et al., 2016; Eriksson et al., 2019). Specifically, the 2015 wave of TIMSS^2^ included the item “My teacher tells me how to do better when I make a mistake.” This allows us to estimate the association between the use of mistake-based feedback and student achievement within each country that participated in 2015 TIMSS.

We shall assume that these associations reflect the effects of mistake-based feedback on achievement (other possibilities are addressed in the discussion). Under this assumption our hypotheses can be tested by examination of how effects of mistake-based feedback correlate with available country-measures of collectivism, power distance, and religiosity.

## Materials and Methods

In brief, the method of our study consists of two steps. The first step is to use TIMSS data to obtain estimates per country of the effect of mistake-based feedback on achievement. The second step is to examine if these estimates are related to country measures of power distance, religiosity, and collectivism.

Out of 55 countries that participated in TIMSS 2015 (4th grade, 8th grade, or both)^3^, we study 49 countries for which country measures of religiosity, power distance, and collectivism were available. The 49 countries are listed in Table 1, which also reports the size of the TIMSS student sample and the number of classes sampled in each country.

**TABLE 1:** TIMSS Sample Sizes and Country Measures from Other Sources than TIMSS.

	Grade 4		Grade 8		Relig. imp.	Power dist.	Collect.	GNI/cap.
Country	students	classes	students	classes				
Australia	6057	498	10338	645	0.32	36	10	43
Belgium (Flemish)	5404	295			0.33	61	22	41
Bulgaria	4228	233			0.34	70	70	16
Canada	12283	696	8757	409	0.42	39	20	43
Chile	4756	179	4849	173	0.70	63	77	22
Croatia	3985	223			0.70	73	67	20
Czechia	5202	265			0.21	57	42	28
Denmark	3710	194			0.19	18	26	45
Egypt			7822	215	0.97	70	75	10
Finland	5015	300			0.28	33	37	39
France	4873	273			0.30	68	29	38
Germany	3948	213			0.40	35	33	45
Hong Kong	3600	145	4155	145	0.24	68	75	54
Hungary	5036	241	4893	241	0.39	46	20	23
Indonesia	4025	312			0.99	78	86	10
Iran	3823	291	6130	251	0.73	58	59	16
Ireland	4344	214	4704	204	0.54	28	30	44
Israel			5512	200	0.51	13	46	31
Italy	4373	257	4481	230	0.72	50	24	34
Japan	4383	148	4745	147	0.24	54	54	37
Jordan			7865	260	0.96	70	70	10
Korea	4669	188	5309	170	0.43	60	82	35
Kuwait	3593	294	4503	191	0.91	90	75	76
Lebanon			3873	185	0.87	75	60	13
Lithuania	4529	290	4347	252	0.42	42	40	26
Malaysia			9726	326	0.96	100	74	25
Malta			3817	223	0.86	56	41	29
Morocco	5068	374	13035	375	0.97	70	54	7
Netherlands	4515	223			0.33	38	20	46
New Zealand	6322	459	8142	377	0.33	22	21	33
Norway	4329	222	4697	216	0.21	31	31	68
Poland	4747	254			0.75	68	40	24
Portugal	4693	321			0.72	63	73	26
Qatar	5194	224	5403	238	0.95	93	75	130
Russia	4921	217	4780	221	0.34	93	61	23
Saudi Arabia	4337	189	3759	149	0.93	95	75	51
Serbia	4036	192			0.54	86	75	12
Singapore	6517	358	6116	334	0.70	74	80	78
Slovakia	5773	327			0.47	100	48	27
Slovenia	4445	255	4257	217	0.47	71	73	29
South Africa			12514	328	0.85	49	35	12
Spain	7764	379			0.49	57	49	33
Sweden	4142	211	4090	206	0.17	31	29	46
Taiwan	4291	177	5711	191	0.45	58	83	46
Thailand			6482	213	0.97	64	80	15
Turkey	6456	251	6079	220	0.82	66	63	19
United Arab Emirates	21177	891	18012	763	0.91	90	75	66
United Kingdom	7122	242	4814	213	0.27	35	11	38
United States	10029	497	10221	534	0.69	40	9	53
Total sample	227714	12012	223938	9262				

*The last four columns are (1) the proportion of the population that thinks religion is important, (2) Hofstede’s measure of power distance, (3) Hofstede’s measure of collectivism, and (4) GNI per capita in thousands of international dollars.*

### Countries, TIMSS Samples, and Country Measures From Other Sources

From the 2009 global Gallup we obtained country measures of *religiosity* in terms of the percentage of the sampled population who responded “yes” to the question: “Is religion important in your daily life?” (Crabtree, 2010). In our sample of countries, the percentage who judged religion as important ranged from 17 to 99 (M = 58, SD = 27).

Estimates of the *power distance* and *individualism* for each country in our sample, on a scale from 0 to 100, were taken from Hofstede’s website^4^ and are reported in Table 1. To obtain a collectivism measure we reverse coded the individualism measure (i.e., collectivism = 100 - individualism). In our sample of countries, power distance ranged from 13 to 100 (M = 59, SD = 22) and collectivism ranged from 9 to 86 (M = 51, SD = 24).

Country scores of *gross national income (GNI) per capita* in 2015, measured in international dollars, were downloaded from the Human Development Report Office of the United Nations^5^. For Taiwan we used the measure for 2015 from their National Statistics agency^6^. In our sample of countries, GNI per capita ranged from 7,000 in Morocco to 130,000 in Qatar (M = 35,000, SD = 22,000).

### Estimation of the Effect of Mistake-Based Feedback on Student Achievement

To estimate the effectiveness of mistake-based feedback we used data from TIMSS on student achievement and teachers’ use of mistake-based feedback, as well as some control variables.

#### Student Achievement in Mathematics and Science

TIMSS uses an elaborate method to measure student achievement in mathematics and science (Martin et al., 2016). In brief, each student responds to only a subset of test questions and five “plausible values” for the total score of each student are generated through an imputation method. Plausible values are given on a scale that was calibrated so that the 1995 TIMSS results had a mean of 500 and a standard deviation of 100. We used the set of five plausible values of student achievement in math and science as measured in TIMSS 2015, standardized within each country to unit standard deviation.

#### Use of Mistake-Based Feedback

The grade 4 and grade 8 student questionnaires of TIMSS 2015 included one part about mathematics and one part about science. Both parts included a set of ten items about the teacher. For each item, students gave their response on a four-point scale: *Disagree a lot* (coded 1); *Disagree a little* (coded 2); *Agree a little* (coded 3); *Agree a lot* (coded 4). Our focus is on the item “My teacher tells me how to do better when I make a mistake,” which we shall refer to as MBF (mistake-based feedback). On the MBF item, almost all responses were either *Agree a lot* or *Agree a little* (93% in grade 4, 85% in grade 8). This means that MBF was nearly a binary variable. (Indeed, if we recode it as binary by lumping the two *Disagree* options together with *Agree a little*, all the main results presented in this paper would remain virtually identical).

Following prior research we average responses from all students in a class to obtain a measure of the teacher’s teaching style (Eriksson et al., 2019). (In the binary recoding, the class average would simply reflect how frequently students in a class responded by *Agree a lot* to the MBF item about a given teacher.) This is taken as a measure of how much the teacher uses mistake-based feedback. The class-average response to the MBF item for each of the two teachers yielded two class-level measures, which we refer to as MBF:Math and MBF:Science. For descriptive statistics of MBF:Math and MBF:Science in each grade in each country, see Table 2. There were eight countries in which science grade 8 was not taught by a single teacher but by several teachers specializing in different science disciplines. For these countries no MBF:Science measures in grade 8 were calculated (as they would be ambiguous).

**TABLE 2:** Descriptive Statistics of MBF Measures.

	Grade 4				Grade 8			
	Math		Science		Math		Science	
Country	Mean	*SD*	Mean	*SD*	Mean	*SD*	Mean	*SD*
Australia	3.58	0.24	3.50	0.26	3.13	0.43	3.02	0.39
Belgium (Flemish)	3.65	0.20	3.52	0.25				
Bulgaria	3.85	0.17	3.84	0.17				
Canada	3.65	0.22	3.59	0.24	3.33	0.34	3.17	0.38
Chile	3.68	0.23	3.65	0.23	3.34	0.40	3.33	0.33
Croatia	3.65	0.20	3.67	0.21				
Czechia	3.42	0.32	3.39	0.32				
Denmark	3.57	0.25	3.36	0.37				
Egypt					3.52	0.26	3.54	0.24
Finland	3.66	0.22	3.60	0.25				
France	3.54	0.22	3.43	0.30				
Germany	3.60	0.26	3.55	0.27				
Hong Kong	3.37	0.26	3.37	0.26	3.12	0.35	3.12	0.32
Hungary	3.68	0.23	3.65	0.23	3.18	0.41		
Indonesia	3.77	0.28	3.72	0.27				
Iran	3.74	0.23	3.77	0.22	3.40	0.35	3.41	0.34
Ireland	3.73	0.20	3.63	0.25	3.28	0.27	3.12	0.36
Israel					3.24	0.36	3.08	0.49
Italy	3.63	0.21	3.59	0.24	3.22	0.36	3.10	0.38
Japan	3.29	0.29	3.17	0.30	2.92	0.29	2.79	0.30
Jordan					3.60	0.22	3.58	0.26
Korea	3.13	0.36	3.06	0.36	2.67	0.27	2.58	0.29
Kuwait	3.67	0.30	3.71	0.29	3.41	0.34	3.42	0.35
Lebanon					3.51	0.34		
Lithuania	3.75	0.16	3.74	0.17	3.37	0.31		
Malaysia					3.44	0.27	3.44	0.29
Malta					3.31	0.38		
Morocco	3.73	0.32	3.74	0.31	3.47	0.33		
Netherlands	3.51	0.24	3.47	0.23				
New Zealand	3.58	0.23	3.49	0.27	3.15	0.36	3.13	0.35
Norway	3.71	0.19	3.65	0.23	3.28	0.37	3.16	0.40
Poland	3.39	0.31	3.39	0.30				
Portugal	3.89	0.13	3.86	0.16				
Qatar	3.61	0.26	3.61	0.25	3.26	0.36	3.24	0.37
Russia	3.78	0.17	3.75	0.18	3.48	0.28		
Saudi Arabia	3.65	0.27	3.60	0.27	3.39	0.34	3.38	0.35
Serbia	3.81	0.15	3.80	0.15				
Singapore	3.51	0.25	3.43	0.28	3.23	0.30	3.20	0.27
Slovakia	3.65	0.26	3.59	0.29				
Slovenia	3.58	0.23	3.57	0.23	3.13	0.29		
South Africa					3.57	0.23	3.46	0.25
Spain	3.81	0.22	3.77	0.24				
Sweden	3.55	0.22	3.44	0.24	3.12	0.35		
Taiwan	3.51	0.28	3.44	0.29	3.20	0.33	3.03	0.32
Thailand					3.48	0.22	3.44	0.24
Turkey	3.82	0.17	3.83	0.17	3.63	0.27	3.61	0.28
United Arab Emirates	3.61	0.27	3.60	0.29	3.33	0.34	3.26	0.34
United Kingdom	3.73	0.17	3.61	0.23	3.28	0.39	3.13	0.29
United States	3.68	0.20	3.61	0.22	3.27	0.39	3.22	0.38

*Each MBF measure is the class-mean response on whether the teacher (math or science) uses mistake-based feedback, on a scale from 1 (disagree a lot) to 4 (agree a lot).*

**FIGURE 1:**
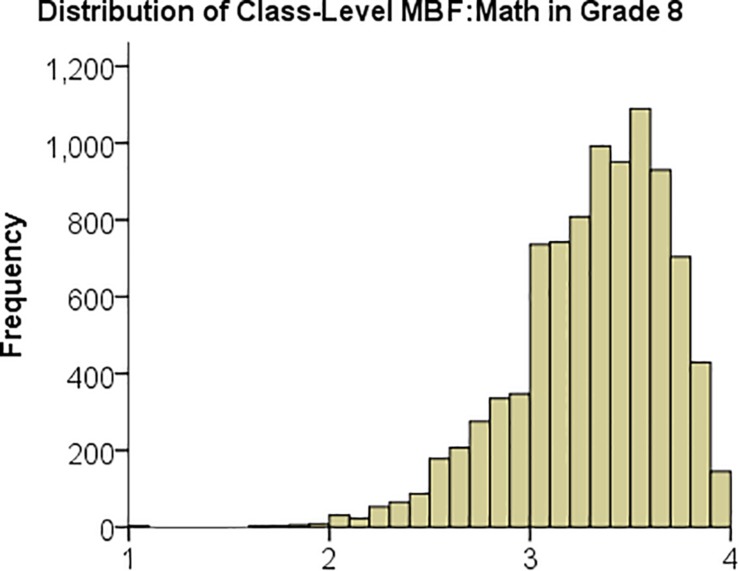
Histogram over MBF:Math, the class-level measure of mistake-based feedback in mathematics, of all classes in 8th grade.

In Table 2, note that the country-means of MBF:Math and MBF:Science are consistently between 3 and 4, reflecting that these were the dominant individual responses. However, there were specific classes where the MBF measures were much lower than 3, as illustrated in Figure 1 showing the distribution of the MBF measure for mathematics across all participating classes in 8th grade. The corresponding distributions for 4th grade mathematics and for science look similar.

#### Control Variables

As described in detail below, we estimate the effect of mistake-based feedback both with and without including control variables. Ideally, results are robust to the model specification. The following control variables are used.

First, when estimating the effect of MBF of the mathematics, we control for the MBF of the science teacher, and vice versa.

In addition to the MBF item, the student questionnaire included nine other items (using the same response scale) about the teacher: “I know what my teacher expects me to do,” “My teacher is easy to understand,” “I am interested in what my teacher says,” “My teacher gives me interesting things to do,” “My teacher has clear answers to my questions,” “My teacher is good at explaining mathematics,” “My teacher lets me show what I have learned,” “My teacher does a variety of things to help us learn,” “My teacher listens to what I have to say.” Note that all of these items are positive statements about the teacher. For each teacher subject (math and science) we calculated the student’s mean response to these items (Cronbach’s α > 0.86 for each academic subject in each grade), and then averaged this measure over all students of the class. We refer to these class-level measures as Pos:Math and Pos:Science. These measures were typically between 3 and 4, meaning that students tended to agree at least a little with the nine positive statements about the teacher. We use the Pos measures as control variables to ascertain that estimated effects of MBF do not simply reflect effects of a generally positive view of the teacher.

When studying antecedents of student achievement it is common to control for socio-economic status and gender. Following some previous research on TIMSS data (Blömeke et al., 2016; Eriksson et al., 2019), we used the response to the item “About how many books are there in your home?” as a proxy for socio-economic status, henceforth referred to as *SES*. This item has a five-point response scale from *None or very few* (*0–10 books*) (coded 1) to *Enough to fill three or more bookcases* (*more than 200*) (coded 5). Student *gender* was coded 1 for girl and 2 for boy.

TIMSS also includes four teacher background variables that we used as controls: experience (years of teaching), age, gender, and level of formal education.

#### Missing Data

There were at most a few percent missing data on the items we use. Missing data were handled using the multiple imputation functionality of SPSS v. 24, generating five sets of imputed data, to each of which one of the five pairs of plausible values on mathematics and science achievement was assigned.

#### Estimation of Within-Country Effects of MBF on Achievement in Math and Science in Grades 4 and 8

To account for the multiple levels of data in each country (class and student) we include a random effect of class (O’Connell and McCoach, 2008). We estimate the effect of MBF *in a given country for a given subject in a given grade* using two different models: without control variables,

$$Y_{\mathrm{ij}}=\gamma_{00}+\gamma_{01}\,{\mathrm{MBF}}_{j}+u_{j}+r_{\mathrm{ij}}$$

and with control variables,

$$Y_{\mathrm{ij}}=\gamma_{00}+\gamma_{01}\,{\mathrm{MBF}}_{j}+\gamma_{02}\,{\mathrm{Pos}}_{j}+\gamma_{03}\,{\mathrm{Ex}}_{j}+\gamma_{04}\,{\mathrm{Ag}}_{j}+\gamma_{05}\,{\mathrm{Ge}}_{j}+\gamma_{06}\,{\mathrm{Ed}}_{j}+\gamma_{07}\,{\mathrm{OMBF}}_{j}+\gamma_{10}\,{\mathrm{SES}}_{\mathrm{ij}}+\gamma_{20}\,{\mathrm{Gen}}_{\mathrm{ij}}+u_{j}+r_{\mathrm{ij}}.$$

Here *Y*_*ij*_ denotes the achievement in the given subject for student *i* in class *j*; γ_00_ is the class-level intercept; *MBF*_*j*_, *Pos_j_*, Ex_*j*_, *Ag*_*j*_, *Ge*_*j*_, and *Ed*_*j*_ are the MBF and Pos measures and the experience, age, gender and level of education for the teacher in the given subject in class *j; OMBF_*j*_* denotes the MBF measure of the teacher in the *other* subject in class *j; SES*_*ij*_ and *Gen*_*ij*_ denote the socio-economic status and gender of student *i* in class *j*; *u*_*j*_ is a random error term representing a unique effect associated with class *j* and *r*_*ij*_ is a random error term at the individual level. Error terms are assumed to have a normal distribution with a mean of zero and constant variance.

Analyses were conducted using restricted maximum likelihood (REML) estimation in the linear mixed model function of SPSS v. 24. Using the SPSS functionality for analysis of multiply imputed data, analyses were performed on each set of imputed data and then pooled to yield unbiased estimates of effects and standard errors.

By the above procedure the effect of MBF on achievement (i.e., the coefficient γ_01_) was estimated up to eight times per country: two models (with controls and without controls) for each of two subjects (math and science) in each of two grades (4th and 8th). Estimates and standard errors are reported in Tables 3, 4. To obtain approximate 95% confidence intervals, take the estimate plus/minus two standard errors.

**TABLE 3:** Estimates of the MBF effect on achievement in Grade 4.

	Math				Science			
	W/o controls		With controls		W/o controls		With controls	
Country	Estimate	SE	Estimate	SE	Estimate	SE	Estimate	SE
Australia	0.02	0.03	0.01	0.03	–0.03	0.02	–0.05	0.03
Belgium (Flemish)	−0.08^∗^	0.03	–0.03	0.04	–0.13^∗∗∗^	0.03	–0.10	0.05
Bulgaria	0.09	0.05	–0.08	0.08	0.13^∗^	0.05	–0.01	0.07
Canada	0.00	0.02	0.02	0.04	–0.01	0.02	−0.13^∗^	0.04
Chile	0.06	0.05	0.00	0.09	0.05	0.05	0.17	0.10
Croatia	0.02	0.03	0.03	0.05	0.03	0.03	0.07	0.05
Czechia	–0.05	0.03	–0.05	0.05	–0.02	0.03	0.04	0.05
Denmark	0.03	0.04	–0.04	0.05	–0.01	0.04	−0.15^∗^	0.07
Finland	–0.02	0.03	–0.01	0.06	–0.01	0.05	0.00	0.09
France	−0.07^∗^	0.03	–0.08	0.04	−0.08^∗^	0.03	–0.05	0.05
Germany	0.05	0.04	0.06	0.05	0.00	0.04	–0.02	0.06
Hong Kong	0.01	0.06	−0.19^∗^	0.09	0.08	0.05	–0.18	0.10
Hungary	–0.06	0.04	0.02	0.06	–0.10	0.04	–0.18^∗∗^	0.06
Indonesia	0.18^∗∗∗^	0.04	0.16^∗^	0.06	0.26^∗∗∗^	0.04	0.25^∗∗∗^	0.06
Iran	0.05	0.04	0.02	0.06	0.04	0.04	–0.02	0.06
Ireland	–0.04	0.03	0.04	0.04	–0.03	0.03	0.01	0.06
Italy	0.01	0.03	–0.02	0.05	0.03	0.03	0.06	0.05
Japan	0.05	0.03	0.03	0.05	0.03	0.03	–0.03	0.07
Korea	0.02	0.03	−0.29^∗^	0.07	0.04	0.04	−0.20^∗^	0.07
Kuwait	0.09^∗^	0.04	0.09	0.07	0.12^∗∗∗^	0.03	0.05	0.06
Lithuania	0.07^∗^	0.03	0.06	0.04	0.08	0.04	0.07	0.05
Morocco	0.14^∗∗∗^	0.04	0.04	0.06	0.10^∗∗^	0.03	0.00	0.05
Netherlands	0.00	0.03	0.09	0.05	–0.05	0.03	−0.17^∗^	0.06
New Zealand	–0.09^∗∗^	0.03	–0.01	0.04	−0.11^∗^	0.04	–0.11	0.07
Norway	0.01	0.03	–0.08	0.04	0.01	0.03	–0.06	0.05
Poland	–0.06	0.03	–0.10	0.05	–0.05	0.03	–0.06	0.05
Portugal	0.02	0.03	–0.03	0.04	0.02	0.03	–0.03	0.04
Qatar	0.31^∗∗∗^	0.04	0.25^∗^	0.09	0.32^∗∗∗^	0.04	0.19^∗^	0.07
Russia	–0.03	0.05	−0.18^∗^	0.07	0.00	0.05	0.11	0.06
Saudi Arabia	0.26^∗∗∗^	0.05	0.21	0.11	0.26^∗∗∗^	0.05	0.15	0.09
Serbia	–0.10^∗∗^	0.03	–0.06	0.05	–0.09^∗∗^	0.03	0.01	0.06
Singapore	0.00	0.04	–0.35^∗∗∗^	0.06	–0.02	0.04	−0.21^∗^	0.08
Slovakia	–0.12^∗∗^	0.04	0.04	0.06	−0.11^∗^	0.04	0.04	0.06
Slovenia	0.00	0.03	–0.09	0.05	0.05	0.03	0.05	0.05
Spain	0.02	0.03	0.08	0.05	–0.02	0.03	–0.12	0.07
Sweden	−0.10^∗^	0.04	–0.20^∗∗^	0.06	–0.07	0.04	–0.03	0.06
Taiwan	0.02	0.03	0.00	0.05	0.02	0.03	–0.09	0.05
Turkey	0.28^∗∗∗^	0.04	0.06	0.05	0.26^∗∗∗^	0.04	0.02	0.05
United Arab Emirates	0.31^∗∗∗^	0.02	0.18^∗∗^	0.06	0.33^∗∗∗^	0.02	0.18^∗∗^	0.06
United Kingdom	0.00	0.01	−0.08^∗^	0.03	0.03^∗∗^	0.01	–0.02	0.04
United States	0.01	0.03	−0.12^∗^	0.05	0.00	0.03	–0.17^∗∗∗^	0.04

*Estimates are standardized coefficients for the effect of MBF on achievement. Stars indicate p-values with respect to the null hypothesis that the true MBF effect is zero (^∗^*p* < 0.05; ^∗∗^*p* < 0.01; ^∗∗∗^*p* < 0.001).*

**TABLE 4:** Estimates of the MBF effect on achievement in Grade 8.

	Math				Science			
	W/o controls		With controls		W/o controls		With controls	
Country	Estimate	SE	Estimate	SE	Estimate	SE	Estimate	SE
Australia	0.14^∗∗∗^	0.03	0.00	0.06	0.08^∗^	0.03	–0.19^∗∗∗^	0.05
Canada	0.02	0.03	−0.16^∗^	0.07	0.00	0.03	–0.29^∗∗∗^	0.07
Chile	–0.03	0.06	–0.21	0.14	0.01	0.05	−0.38^∗^	0.15
Egypt	0.03	0.04	0.23^∗^	0.09	0.10^∗^	0.04	0.05	0.09
Hong Kong	0.08	0.07	0.23	0.20	0.09	0.06	−0.45^∗^	0.18
Hungary	–0.10	0.05						
Iran	–0.01	0.04	0.08	0.10	0.00	0.04	0.04	0.10
Ireland	0.00	0.04	−0.15^∗^	0.07	0.13^∗∗^	0.04	–0.07	0.07
Israel	–0.10	0.05	–0.32^∗∗^	0.11		0.05	−0.37^∗^	0.14
Italy	–0.03	0.03	–0.07	0.08	−0.07^∗^	0.03	–0.12	0.08
Japan	0.07^∗^	0.03	–0.03	0.07	0.07^∗^	0.03	−0.14^∗^	0.06
Jordan	0.08^∗^	0.03	0.14	0.07	0.09^∗∗^	0.03	0.08	0.07
Korea	–0.03	0.03	–0.16^∗∗^	0.05	0.10^∗∗∗^	0.02	–0.09	0.05
Kuwait	0.03	0.04	–0.05	0.08	0.13^∗^	0.05	–0.01	0.12
Lebanon	0.12^∗^	0.05						
Lithuania	0.02	0.04						
Malaysia	0.41^∗∗∗^	0.05	0.43^∗∗∗^	0.11	0.50^∗∗∗^	0.04	0.46^∗∗∗^	0.11
Malta	0.17^∗∗^	0.06						
Morocco	0.05	0.03						
New Zealand	0.03	0.04	–0.06	0.08	0.07	0.04	–0.15	0.08
Norway	0.08^∗^	0.03	–0.06	0.05	0.03	0.03	0.03	0.08
Qatar	0.21^∗∗∗^	0.04	0.11	0.10	0.20^∗∗∗^	0.04	–0.15	0.11
Russia	0.07	0.04						
Saudi Arabia	0.11^∗^	0.05	0.03	0.13	0.15^∗∗^	0.05	0.05	0.16
Singapore	0.08	0.05	–0.34^∗∗^	0.10	–0.03	0.05	–0.43^∗∗∗^	0.10
Slovenia	0.00	0.03						
South Africa	–0.08	0.04	0.20^∗∗^	0.07	–0.15^∗∗∗^	0.04	−0.23^∗^	0.08
Sweden	0.01	0.04						
Taiwan	0.04	0.04	–0.17^∗∗^	0.06	0.07^∗^	0.03	–0.11	0.06
Thailand	–0.10	0.06	0.25^∗^	0.12	0.05	0.06	0.23^∗^	0.11
Turkey	0.06	0.04	0.08	0.07	0.09^∗^	0.04	0.14	0.09
United Arab Emirates	0.23^∗∗∗^	0.02	0.17^∗^	0.07	0.18^∗∗∗^	0.02	0.09	0.08
United Kingdom	0.04	0.07	–0.60^∗∗∗^	0.14	0.12	0.06	0.00	0.11
United States	0.03	0.03	–0.27^∗∗∗^	0.07	0.05	0.03	–0.33^∗∗∗^	0.07

*Estimates are standardized coefficients for the effect of MBF on achievement. Stars indicate p-values with respect to the null hypothesis that the true MBF effect is zero (^∗^*p* < 0.05; ^∗∗^*p* < 0.01; ^∗∗∗^*p* < 0.001). For eight countries in which science is taught by several teachers, no MBF measure for science was calculated. Hence for these countries we could not estimate the MBF effect for science, nor could we estimate the MBF effect for math with controls (as the MBF for science is one of the controls in the model).*

### An Alternative Approach

Our main approach has two steps. In the first step we estimate the effect of class-level MBF on student achievement separately in each country, using a two-level analysis (student and class). In the second step we examine how these estimates per country relate to country-level measures of power distance, religiosity, and collectivism.

An alternative approach is to include all countries from the beginning in a three-level analysis (student, class, and country) of student achievement to examine the interaction of class-level MBF and country-level measures. Without controls, the model for a given subject in a given grade would then be

Yijk=γ000+γ001⁢CLMk+γ011⁢MBFjk+γ012⁢CLMk⁢MBFjk*+vk+ujk+rijk,

where *Y*_*ijk*_ denotes the achievement in the given subject for student *i* in class *j* in country *k*; γ_000_ is the country-level intercept; *CLM*_*k*_ is a country-level measure (say, power distance) in country *k*; *MBF*_*jk*_ is the MBF measure for the teacher in the given subject in class *j* in country *k; v_*k*_* and *u*_*jk*_ are random error terms representing unique effects associated with country *k* and class *j*, respectively, and *r_ijk_* is a random error term at the student level. When adding control variables to this model, we also include their interactions with the country-level measure (e.g., we would include a *Pos*_*jk*_ term as well as the interaction term *CLM*_*k*_^∗^*Pos*_*jk*_).

The advantage of our main approach is that we explicitly obtain country estimates of the MBF effect, thereby allowing easy examination of their consistency across grades, subjects, and model specifications, as well as easy illustration of their relation to a country measure using a scatter plot. The advantage of the alternative approach is that it yields a more accurate estimate of the statistical significance of the latter relation, which in the above model is captured by the interaction term *CLM*_*k*_^∗^*MBF*_*jk*_. We use the alternative approach only to verify the statistical significance of the interaction. These analyses were performed in the lme4 package (Bates et al., 2014).

## Results

### Descriptive Statistics of Estimated MBF Effects

In the estimation of MBF effects, all variables were standardized within each country. Therefore, estimated MBF effects are measured in the unit “within-country standard deviation in achievement per within-country standard deviation in MBF.”

Starting with grade 4, the estimated MBF effects per country in Table 3 can be summarized as follows: The mean MBF effect was close to zero in both subjects, regardless of model specification, but there was substantial variation between countries. To illustrate, consider MBF effects for math estimated with controls in grade 4: the mean effect was −0.01, *p* = 0.47, with a standard deviation of 0.12 and a range from −0.35 to 0.25. Thus, it seems that there are some countries where the MBF effect is positive and other countries where the MBF effect is negative.

Estimated MBF effects per country in grade 8 showed the same pattern, see Table 4. To illustrate, consider MBF effects for math estimated with controls in grade 8: the mean effect was −0.03, *p* = 0.56, with a standard deviation of 0.22 and a range from −0.60 to 0.43.

### Relation Between the MBF Effect and Other Country Variables

Table 5 reports pairwise correlations, with bootstrapped confidence intervals, of the estimated MBF effects against religiosity, power distance, collectivism, and GNI per capita. The table reveals a consistent pattern. Regardless of the method and data used to estimate the MBF effect, it was always positively correlated with religiosity, power distance, and collectivism. The strength of the correlations varied across different estimates, but overall correlations tended to be stronger for religiosity (average correlation = 0.47) and power distance (average correlation = 0.44) than for collectivism (average correlation = 0.33). In Table 5, a few of the confidence intervals include zero, indicating a non-significant relation. However, when we conducted corresponding analyses using the alternative approach described in section 2.3, the interaction between MBF and these culture variables always came out as significantly positive. Thus, we conclude that there are robust positive associations between MBF effects on the one hand and religiosity, power distance, and collectivism on the other hand.

**TABLE 5:** Correlations between MBF effect estimates and country measures from other sources.

MBF Effect Estimates			Religion important	Power distance	Collectivism	GNI per capita
Grade	Subject	Controls				
4th	Math	without	0.66[0.43,0.77]	0.38[0.04,0.65]	0.46[0.26,0.62]	0.31[−0.19,0.63]
		with	0.46[0.12,0.71]	0.18[−0.17,0.53]	0.05[−0.28,0.43]	0.13[−0.44,0.54]
	Science	without	0.65[0.42,0.77]	0.46[0.14,0.72]	0.57[0.39,0.71]	0.28[−0.25,0.61]
		with	0.52[0.24,0.71]	0.51[0.28,0.69]	0.41[0.07,0.72]	−0.02[−0.53,0.34]
8th	Math	without	0.26[−0.09,0.51]	0.54[0.24,0.72]	0.23[−0.12,0.48]	0.33[−0.02,0.67]
		with	0.50[0.18,0.75]	0.54[0.26,0.75]	0.41[0.03,0.69]	−0.18[−0.56,0.11]
	Science	without	0.24[−0.15,0.50]	0.53[0.15,0.74]	0.31[0.06,0.53]	0.18[−0.24,0.65]
		with	0.45[0.10,0.69]	0.41[0.04,0.67]	0.23[−0.12,0.54]	−0.25[−0.53,0.01]

*Correlations are calculated based on 41 data points (countries) in grade 4, based on 34 data points in 8th grade math without controls, and based on 26 data points in the other three analyses of 8th grade data. Correlation coefficients are reported with 95% confidence intervals (BCa, 1000 bootstrap samples).*

To increase the set of countries and use both grades and both subjects, we calculated an aggregate estimate of the controlled MBF effect by taking the average of all available controlled estimates for a given country. This yielded an aggregate estimate of the controlled MBF effect for 47 different countries. This aggregate estimate correlated with religiosity at *r* = 0.54, bootstrapped 95% CI [0.30,0.73], with power distance at r = 0.52 [0.24,0.71], and with collectivism at *r* = 0.37 [0.08,0.63]. Using religiosity, power distance, and collectivism as simultaneous predictors in a multiple linear regression of the aggregate MBF effect, we found they together explained 37% of the total variance, with statistically significant independent effects of both religiosity, β = 0.38, *p* = 0.015, and power distance, β = 0.39, *p* = 0.035, but not of collectivism, β = −0.11, *p* = 0.52.

**FIGURE 2:**
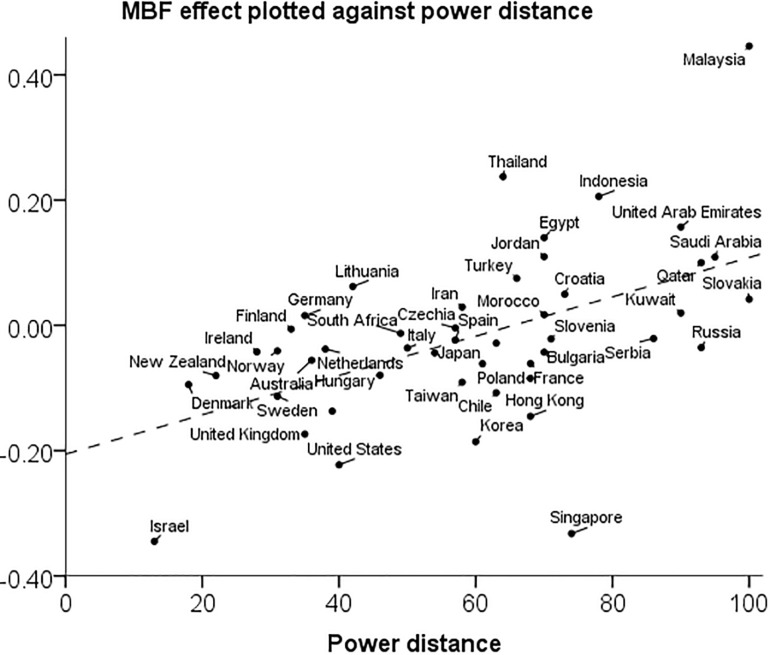
Scatter plot of the aggregate controlled MBF effect against power distance in 47 countries.

The relation between the aggregate MBF effect and power distance is illustrated by a scatter plot in Figure 2. Note that the regression line fits the data points fairly with two exceptions: Singapore and Malaysia are outliers in different directions. If the two outliers are excluded, the correlation between the MBF effect and power distance increases slightly to *r* = 0.55 [0.38,0.71], and similarly for the correlations with religiosity, *r* = 0.58 [0.32,0.78] and collectivism, *r* = 0.46 [0.19,0.72].

## Discussion

In this paper we have used data from an international assessment of mathematics and science achievement to examine the effect of teachers giving feedback on students’ mistakes. This is a teaching practice that has both proponents and critics. Our data support both views. In some countries (such as the United Arab Emirates) we found a positive association between teachers’ use of feedback on mistakes and their students’ achievement relative other students in the same country. In some other countries (such as the United States), the association was negative, at least after controlling for some potential confounders.

Based on prior cross-cultural work on negative feedback in other contexts (e.g., Heine et al., 2001a, b), we hypothesized that culture would moderate the effectiveness of mistake-based feedback. Specifically, it should be more effective in cultures where teachers have more authority. In the absence of a direct measure we examined two other widely available cultural measures, power distance and religiosity, which other scholars have thought to be associated with teachers’ authority (e.g., Hofstede, 1986; Smith, 2013). In line with our hypothesis, we found both measures to be positively correlated with the effectiveness of mistake-based feedback.

We also hypothesized that mistake-based feedback would be more effective in cultures where students are more motivated to adapt to consensually shared standards and are more likely to have a growth mind-set. In the absence of direct measures we examined another widely available cultural measure, collectivism, which other scholars have thought to be associated with these traits (e.g., Littlewood, 1999; Gelfand et al., 2002). Although collectivism was indeed found to be associated with the effect of mistake-based feedback, this association disappeared when we controlled for power distance and religiosity. For this reason we tentatively conclude that teachers’ authority is the main moderator of the effectiveness of mistake-based feedback.

When drawing conclusions from our study, some important limitations must be acknowledged. First, to measure the use of mistake-based feedback we only had access to a single student questionnaire item with a simple four-step scale (on which the vast majority of students used only the third or fourth step). A more complex measure would have been preferable for two reasons. For one thing, mistake-based feedback is a complex phenomenon, the many nuances of which a single item is unable to capture. For another, a single-item measure will typically have poor reliability. A likely consequence of poor reliability of the MBF measure is that the size of MBF effects on achievement will tend to be underestimated. In other words, with a more reliable measure of MBF we should expect to observe larger effects on achievement.

A second limitation is that our results are purely correlational. Within countries, we have assumed that a certain relation between use of feedback on mistakes and student performance is evidence of the effectiveness of the feedback practice. An alternative possibility is that associations reflect teachers adapting their teaching practices to the performance level of the student group. Under this alternative interpretation, our between-countries finding would require that teachers respond to higher student performance levels by *increasing* the use of feedback on mistakes in high power distance countries, whereas teachers in low power distance countries would respond to high-performers by *decreasing* their use of such feedback. This interpretation, although equally interesting, seems less plausible to us.

As mentioned above, our simple measure do not allow us to distinguish between different ways of implementing mistake-based feedback. There are many ways of using errors as a springboard for further learning (Borasi, 1994; Boaler, 2016). Thus, it is an open question to what extent the difference in effectiveness between countries lies in teachers implementing mistake-based feedback differently and to what extent it lies in students responding differently to the same feedback. Our findings are consistent with the hypothesis that we gave in the introduction: in countries that are high on power distance and religiosity, young people are more accepting of teachers’ authority and therefore more accepting of negative feedback. However, we acknowledge that in the absence of more direct evidence there may be other explanations of the associations we have found.

## Conclusion

Cultural psychologists have long been interested in how negative feedback may work differently in different cultures. Here we have examined how teachers’ feedback on mistakes in math and science class is associated with student achievement in 49 countries. This study differs from classic cross-cultural studies of feedback, both in context and methodology. Still, the finding that feedback on mistakes was associated with better achievement in countries where authority is expected to be more important (namely, countries that are high on power distance and religiosity) was as we expected from prior research. These results highlight the importance of cultural values in educational practice.

## Data Availability Statement

The data files used in the analysis can be accessed in the Open Science Framework data repository (https://osf.io/z3h5c/).

## Ethics Statement

Ethical review and approval was not required for the study on human participants in accordance with the local legislation and institutional requirements. Written informed consent from the participants’ legal guardian/next of kin was not required to participate in this study in accordance with the national legislation and the institutional requirements.

## Author Contributions

KE performed the statistical analysis and wrote the manuscript together with JL. All authors contributed to the conception of the study, read and approved the submitted version. OH and AR provided critical revision inputs.

## Conflict of Interest

The authors declare that the research was conducted in the absence of any commercial or financial relationships that could be construed as a potential conflict of interest.

## Footnotes

1 The teacher’s authoritative role in high power distance societies may at the same time provide a barrier for other teaching practices such as self-evaluation and peer-assessment (Brown et al., 2009; Carless and Lam, 2014; Thanh Pham and Renshaw, 2015).

2 Data and questionnaires are available at https://timssandpirls.bc.edu/timss2015/international-database/.

3 TIMSS data from England and Northern Ireland were merged to represent the United Kingdom.

4 https://www.hofstede-insights.com/country-comparison/

5 http://hdr.undp.org/

6 http://eng.stat.gov.tw/
